# DNA barcoding identification of Greek freshwater fishes

**DOI:** 10.1371/journal.pone.0263118

**Published:** 2022-01-26

**Authors:** Alexandros Tsoupas, Sofia Papavasileiou, Styliani Minoudi, Konstantinos Gkagkavouzis, Olga Petriki, Dimitra Bobori, Argyrios Sapounidis, Emmanouil Koutrakis, Ioannis Leonardos, Nikoleta Karaiskou, Alexandros Triantafyllidis

**Affiliations:** 1 Faculty of Sciences, Department of Genetics, Development and Molecular Biology, Laboratory of Animal Population Genetics, School of Biology, Aristotle University of Thessaloniki, Thessaloniki, Greece; 2 Bioanalysis and Omics (BIOMIC), Center for Interdisciplinary Research and Innovation (CIRI-AUTH), Balkan Center, Thessaloniki, Greece; 3 Faculty of Sciences, Department of Zoology, Laboratory of Ichthyology, School of Biology, Aristotle University of Thessaloniki, Thessaloniki, Greece; 4 Department of Inland Waters and Lagoons, Fisheries Research Institute, Hellenic Agricultural Organization “DEMETER”, Nea Peramos, Kavala, Greece; 5 Department of Biological Applications and Technologies, Laboratory of Zoology, School of Health Sciences, University of Ioannina, Ioannina, Greece; SOUTHWEST UNIVERSITY, CHINA

## Abstract

Biodiversity is a key factor for the functioning and efficiency of an ecosystem. Greece, though covering a relatively small surface area, hosts a great deal of species diversity. This is especially true for freshwater fishes. In recent years, the traditional methods of species identification have been supplemented by the use of molecular markers. The present study therefore aims to extensively produce DNA barcodes for Greek freshwater fish species and investigate thoroughly if the presently accepted species classification is in agreement with molecular data. A 624-bases long fragment of the *COI* gene was sequenced, from 406 freshwater fish specimens belonging to 24 genera and originating from 18 lake and river sites. These sequences were used along with 596 sequences from the same genera, recovered from BOLD, for the construction of phylogenetic trees and the estimation of genetic distances between individuals. In total, 1002 sequences belonging to 72 species were analyzed. The method was found to be effective for 55 of 72 studied species. 17 closely related species with low interspecific genetic distances were observed, for which further study is proposed. It should also be noted that, in four cases, cryptic diversity was observed, where groups originally identified as one species exhibited genetic distance great enough to be separated into discrete species. Region specific haplotypes were also detected within populations of 14 species, giving the possibility to identify even the geographic origin of a species. Our findings are discussed in the light of the rich history of the Balkan peninsula and provide a significant steppingstone for the further study of Greek and European freshwater fish biodiversity.

## 1. Introduction

Biodiversity is a key factor for the functioning and efficiency of an ecosystem [1], while it also has an important role in the production of goods and a major economic impact on human societies [2]. In recent years, a plethora of human activities has led to the destruction of many ecosystems and consequently to the loss of biodiversity with the disappearance of a large number of species [3]. The consequences of this ecological disaster are unpredictable and potentially perilous for humanity [4]. The conservation and management of biodiversity have thus been designated as critical issues [5] and, as biodiversity is directly related to the number of species living on earth [6], species identification is of great importance.

Although Greece has a relatively small surface area (about 132,000 Km^2^), it hosts a great deal of species diversity [7]. This is especially true for freshwater fishes, since 177 species have been recorded in Greece [8], 47 of which are endemic [9]. Almost all the endemic species are protected by National and European legislation (i.e., 92/43/EC). The study and conservation of this diverse ichthyofauna requires the accurate identification of the species present in each region.

The distribution of freshwater fish species in Greece has been shaped by its geographical history and characteristics [10]. Based on the distribution of species, Greece has been divided into biogeographical regions, with the most recent division consisting of four main regions [11], namely the regions northern Aegean, south-eastern Adriatic, Ionian, and western Aegean. Oikonomou et al. [12], based on the distribution of endemic species, proposed the existence of a fifth biogeographical region, which consists of only two lakes, the Mikri Prespa and Megali Prespa Lakes.

In recent years, the traditional methods of species identification have been supplemented by the use of molecular markers [13]. One such approach is DNA barcoding, proposed by Hebert et al. [14], which relies on the sequence of a fragment of the cytochrome c oxidase subunit I (*COI*) gene to separate species and assign individuals to species. The sequences used in this approach are called DNA barcodes and they are submitted to BOLD (http://boldsystems.org/), a database for storing and analyzing DNA barcodes [15].

The efficiency of DNA barcoding has been proved in many studies [16–19], since in most of them more than 90% of the species studied can be accurately identified to species level [20]. DNA barcoding can reveal cryptic diversity [21,22] and identify samples when morphological traits cannot, for example in the case of processed commercial products [23–25]. Additionally, DNA barcoding offers the possibility to detect the presence of species in an area by analyzing environmental DNA, in an approach called DNA metabarcoding [26,27].

DNA barcoding has been successfully used in studies on many groups of animals, such as birds [28], fishes [29], gastropods [30], spiders [31] and Lepidoptera [22,32]. An international campaign for DNA barcoding of freshwater fishes [33] has taken place in many different parts of the world, e.g., America [34–36], Africa [37], Asia [38,39], Australia [29] and Europe [40–42].

DNA barcoding on Greek freshwater fish species has been initially applied by Triantafyllidis et al. [42], who focused on the fish species diversity of four lakes of northern Greece, while Geiger et al. [40] analyzed specimens from various Greek regions through their broader study around the Mediterranean. The present study aims to study the freshwater fish biodiversity of Greece, providing new data on its genetic diversity, not only by analyzing additional samples from already studied sites, but also by studying new, unexplored populations. The results of the two aforementioned studies were also combined with the findings of the present study, providing a more complete picture of the population and species relationships. Additionally, through the DNA sequences, it was even possible to search for region specific haplotypes of the studied species.

## 2. Materials and methods

### 2.1 Sampling

**Fig 1:**
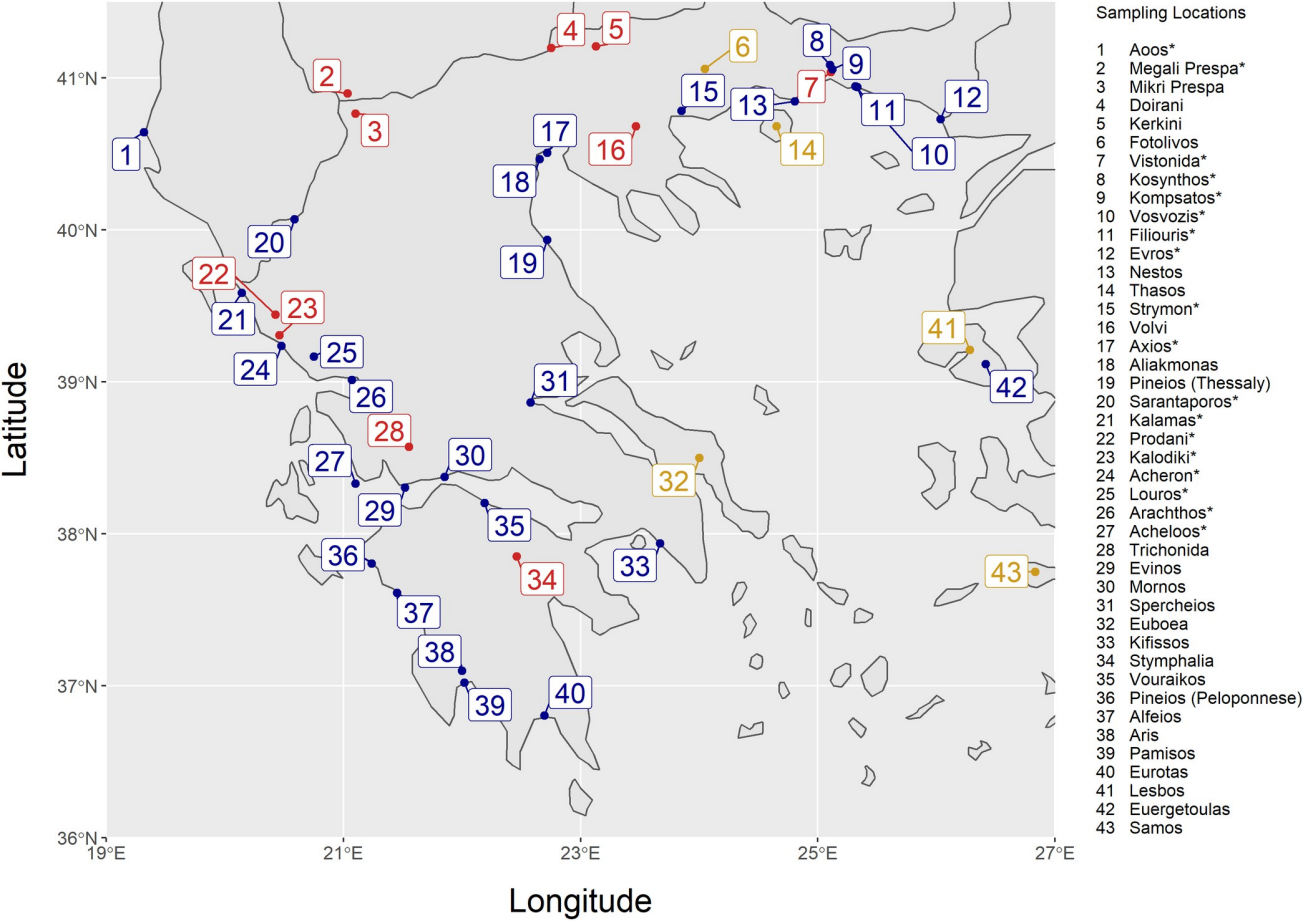
Map of sample origin areas. For the rivers, the location of the mouth is given. Rivers are marked with blue and lakes with red. A location marked with gold corresponds to the name of the broader sampling region and not a specific river or lake. The areas from which samples were gathered during the present study are marked with an asterisk.

544 specimens of Greek freshwater fishes were collected from 18 lake and river sites (Fig 1). All specimens were preserved in 99% ethanol and were initially identified on species level by using morphological traits (S1 Table).

Sampling in river sites was conducted by electrofishing (Hans Grassl ELT60IIHI device) following the European protocol CEN 2003 [43]. Accordingly, specimens from lakes were derived by net sampling, following the European protocol CEN 2005 [44]. During the samplings, all captured fish were identified on site, and after their total length was recorded, they were released back in the water alive. For the Barcoding purposes, at least 2–3 individuals per common species (when available) were preserved in alcohol. In the case of protected species, a small part of the fish, i.e., a fin, was stored for the DNA analysis and the fish was released back to the water alive.

### 2.2 Ethical statement

The samples were collected during faunal monitoring surveys which were conducted in the frame of the implementation of WFD 2000/60 in Greece, covering the period 2010–2015.

All necessary permissions were provided by the Ministry of Environment, Energy & Climate Change (in Greek).

Fish handling and treatment were in accordance with the local guidelines for treating animals which comply to the Official Journal of the Greek Government No. 106/30 April 2013 on the protection of animals used for scientific purposes.

### 2.3 DNA extraction and PCR

DNA was successfully extracted from 406 samples, belonging to 24 genera and 39 species, using the protocol of Hillis et al. [45]. The numbering of the samples, the species in which they belong, and their sampling sites are given in S1 Table. All specimens are stored in the Laboratory of Animal Population Genetics in the School of Biology of the Aristotle University of Thessaloniki. PCR was used for the amplification of a fragment of 624 bases from the 5’ end of the *COI* gene, using the primers FishF2 and FishR2 for *Gambusia holbrooki* Girard, 1859 and the primers FishF1 and FishR1 for all other species [29]. Each PCR reaction had a volume of 22.0 μl, containing 1.1 μl of template DNA, Qiagen Taq DNA polymerase (5.00 units/μl) at a final concentration of 0.04 units/μl, QIAGEN PCR Buffer at a concentration of 1X, a concentration of 1.0 pmol/μl for each primer and 0.2 mM for each deoxynucleotidetriphosphate (dNTP). Reactions were amplified through 35 cycles with the following parameters: initialization step at 95°C for 5 min, denaturation at 94°C for 45 s, annealing at 60°C for 45 s, elongation at 72°C for 90 s and final elongation at 72°C for 7 min.

Sanger sequencing of the DNA fragments was carried out at Genewiz (www.genewiz.com), using ABI 3730xl DNA Analyzers.

### 2.4 Data analysis

The resulting sequences were aligned, and their quality was assessed using the Geneious 10 software [46]. In total, 1002 sequences were used for subsequent analyses (S2 Table), 406 of which were obtained during the present study, while the remaining 596 were retrieved from BOLD (http://boldsystems.org/). The 406 sequences obtained and analyzed in this study were submitted in the BOLD database and the BOLD IDs assigned to them are GFFB001-17 to GFFB055-17, GFFB057-17 to GFFB097-17 and GFFB098-18 to GFFB407-18 (S1 Table). The sequences retrieved from BOLD belonged to the same 24 genera as the sequences of the present study, but they include 33 more species raising the total number of species to 72 (S2 Table) and the total number of sampled sites to 43 (Fig 1). The majority of retrieved sequences belong to Greek specimens, apart from 30 sequences of the genus *Alburnoides*, 7 sequences of the genus *Gobio* that come from various European countries and 1 sequence of the genus *Pseudorasbora* that comes from Turkey. Information (BOLD IDs, species, and area of origin) for the sequences retrieved from BOLD is given in S3 Table. The complete dataset of sequences used during this study is also provided in FASTA format in the Supporting Information.

Phylogenetic trees were constructed for all studied species together as well as for each genus separately, except for *Lepomis gibbosus* (Linnaeus, 1758) and *G*. *holbrooki*. These two species exhibited only one haplotype each and therefore there was not any genetic differentiation on which to base a phylogenetic tree. The phylogenetic trees were constructed with the MEGA7 software [47], using the Maximum likelihood method [48], with Kimura’s two-parameter evolutionary model [49], and applying the bootstrap method [48], set to 1000 replicates for testing the trees [50,51]. For the construction of the phylogenetic trees, the sequences of each genus were truncated, so as sequences produced during this study to have the same length as the ones recovered from BOLD. For the phylogenetic tree of all the species, the sequences were truncated to a length of 598 nucleotides.

Subsequently, through the MEGA7 software [47], the genetic distances in various taxonomic levels were calculated, using Kimura’s two-parameter model and bootstrap of 1000 replicates. We calculated the mean genetic distance within and between species, genera and families, and the mean genetic distance in the total of the sequences. Additionally, we calculated the pairwise genetic distances between the individuals of each genus. From the pairwise distances between individuals of every species and each genus, we were interested in the maximum distance within each species and the minimum distance between species.

Taxonomic changes were proposed by combining the topology of phylogenetic trees with the calculated genetic distances. When the individuals of a single species were grouped into distinct clades, genetic distances were used to assess whether these clades corresponded to populations of the same species or whether each clade could consist a separate species. Additionally, when different species were grouped together on the phylogenetic tree, genetic distances were used to assess if these species could possibly consist a single species. This assessment was made using as a threshold a mean genetic distance of 0.0200, which has also been proposed in other studies [40,52,53]. Groups that had an average genetic distance greater than 0.0200 were proposed as potentially separate species, while for groups that had a mean genetic distance less than 0.0200, further study was proposed, to test whether their merging into a single species would be justified. One additional condition for proposing the taxonomic changes was the presence or absence of a barcoding gap [30]. Initially this was proposed by Hebert et al. [28] for cases that the average genetic distance between groups was ten times the mean distance within each group, in order for these groups to be classified as separate species. However, and since the tenfold magnitude has been questioned [54,55], we considered that a barcoding gap existed when the minimum genetic distance between two groups was greater than the maximum genetic distance within each group [56].

Additionally, we used the Automatic Barcode Gap Discovery (ABGD) analysis [57]. This method uses the pairwise distances between the sequences and the prior of intraspecific divergence to detect a gap between intra and interspecific divergence, which it uses to partition the input samples into groups. The difference from the other approach we used is that ABGD does not use a lower threshold of differentiation to delimit groups and tries to estimate the size of the barcode gap by itself. For the analysis we used the web interface of the software (https://bioinfo.mnhn.fr/abi/public/abgd/abgdweb.html), and the following parameters: Pmin = 0.001, Pmax = 0.1, Steps = 10, X = 1.5 and the Kimura evolutionary model, with TS/TV = 2.0.

## 3. Results and discussion

Barcoding has proven to be a reliable approach for documenting biodiversity at species level. DNA barcoding relies on the comparison of DNA sequences to classify specimens, which means that it relies on the availability of as many sequences as possible, with the broadest geographic distribution possible [58]. As more studies on DNA barcoding are implemented and new populations from more regions are analyzed, the accuracy of the method is improving. By producing additional DNA barcodes, many of which come from new populations, this study contributes to the improvement of the overall DNA barcoding accuracy. Our findings also provide a new insight on Greek biodiversity and valuable knowledge for its management and conservation, since it consists a hot spot for Balkan’s biodiversity [59].

### 3.1 DNA barcoding results

Of the 72 studied species, 55 were successfully identified through DNA barcoding, resulting in a 76.4% success rate of the approach. The genetic distances calculated for each species, as well as information for special cases that are discussed in more detail later, are presented in S4 Table.

The success rate of DNA barcoding in most studies ranges from 76.75% to 100% [34–37,39–41,60–63]. Therefore, the success rate in our study is close to, but slightly lower than that of other published studies.

The 17 species which were not successfully identified had a mean genetic distance from another species of the genus lower than 0.0200 and there was no barcoding gap between them. Three of these species are the studied species of the genus *Salmo*, and the lack of genetic differentiation made it impossible to separate any of these species from the other two. The other 14 species are seven pairs where the two species of each pair cannot be distinguished from one another, but the pair is clearly separated from the other species of the genus. These cases are presented in more detail in part 3.4.

Among the species that were successfully identified, 96.4% exhibited a barcoding gap, the only exception were the species *Barbus euboicus* Stephanidis, 1950 and *Barbus peloponnesius* Valenciennes, 1842. The values of the genetic distances (S4 Table) confirm that only in rare cases the mean interspecific distance is ten times the mean intraspecific distance, so the absence of overlap between minimum interspecific and maximum intraspecific distance seems more efficient as a criterion of barcoding gap.

Apart from the 17 species that were not successfully identified, there were three cases of species with mean genetic distance lower than 0.0200 (S4 Table), which exhibited however, a barcoding gap and could thus be successfully identified. These cases are presented in more detail in part 3.4.

Additionally, in the case of five genera (*Alburnoides*, *Cobitis*, *Gobio*, *Pelasgus*, *Telestes*), geographically separated populations of the same species exhibited a mean genetic distance greater than the threshold of 0.0200 as well as a barcoding gap, which means that according to the criteria we have set, they could be classified as separate species. These cases are presented in more detail in part 3.3.

It should be mentioned that, during the phylogenetic analysis, the clades of the phylogenetic trees were supported by high bootstrap values, exceeding 88, with the exception of the genus *Salmo*, which had bootstrap values as low as 62. This means that in almost all phylogenetic trees the clades are well supported, and they most probably represent the true phylogenetic relationships among the samples. An overview of the relationships of all the different studied species can be seen on the phylogenetic tree of S1 Fig.

### 3.2 Genetic distances at above-species level

The mean genetic distance in the total of the sequences was 0.1710, while within the *Cyprinidae* and *Cobitidae* families, the only families with more than one studied genera, the distance was 0.1410 and 0.1220 respectively (Table 1).

**Table 1:** Mean genetic distance in the total of sequences and mean genetic distance on a family, genus, and species level. In the parentheses is given the standard error of the genetic distance.

Genetic distance	Value (Standard Error)
Mean genetic distance in the total of sequences	0.1710 (0.0120)
Mean genetic distance in the family Cyprinidae	0.1410 (0.0110)
Mean genetic distance in the family Cobitidae	0.1220 (0.0100)
Mean genetic distance on family level	0.1315 (0.0105)
Mean intrageneric genetic distance	0.0320 (0.0043)
Mean intraspecific genetic distance	0.0029 (0.0013)

The intra-family distances were on par with the corresponding values calculated for freshwater species in many other studies (Table 2), slightly lower than the only other study dealing with Greek freshwater species [42], but significantly higher than the one of Geiger et al. [40] that analyzed fish species around the Mediterranean.

**Table 2:** Mean intraspecific and intrageneric genetic distances from broad scale studies. In the first column the area covered by the study is given, followed by the study reference.

Study Region	Mean intraspecific genetic distance	Mean intrageneric genetic distance	Mean intra-family distance genetic distance
Northern Greek lakes [42]	0.0066	0.0380	0.1534
Mediterranean [40]	0.0059	0.0290	0.0644
Germany [41]	0.0026	0.0300	0.1577
America [23,34–36,61,64,65]	0.0017–0.0073	0.0400–0.1380	0.1360–0.2100
Africa [37,66]	0.0017–0.0096	0.1030–0.2070	0.1400 (approximately) - 0.1720
Asia [39,60,67,68]	0.0030–0.0042	0.0600–0.1580	0.1250–0.1630

The mean intrageneric genetic distance in genera including more than one species was found to be 0.0340, close to the corresponding values from studies regarding Europe (Table 2). It is observed that mean intrageneric distances seem to be lower in Europe than in the other continents.

The mean intraspecific genetic distance calculated (0.0029) either approximated or was lower than the corresponding values calculated for freshwater species in many other studies (Table 2). Compared to the two studies that have analyzed samples from Greece [40,42], the value of mean intraspecific distance in our study was lower. It is observed (Table 2) that on a global scale the mean intraspecific genetic distance does not exceed 0.0100, while the distances within a genus are many times greater, which can be used as an argument in favor of the separation of species based on genetic distances.

### 3.3 Species with mean intraspecific genetic distance greater than 0.0200

In the case of five genera (*Alburnoides*, *Cobitis*, *Gobio*, *Pelasgus*, *Telestes*), geographically separated populations of the same species exhibited a mean genetic distance greater than the threshold of 0.0200 and a barcoding gap. These five cases seem to represent cryptic diversity and thus, the splitting of the existing species into additional ones, is proposed. In summary, one separate species is proposed in the genus *Cobitis*, one in the genus *Gobio*, two in the genus *Pelasgus* and two in the genus *Telestes*. It should be noted that we propose the splitting of the existing species into additional ones, which have not been previously described in Greece, without necessarily proposing new candidate species. This would require the comparison of these separate species with all the same species of the genus found outside Greece, a task that exceeds the goal of the present study. Additionally, we propose the assignment of the studied *Alburnoides bipunctatus* (Bloch, 1782) populations to other existing species. More specifically:

### Species of the genus *Alburnoides*

*Alburnoides bipunctatus* is considered a species with broad distribution in Greece, being present in three biogeographical regions (Ionian Sea, Aegean Sea and Southern Adriatic Sea) [9]. However, in the present study, all the populations originally identified as *A*. *bipunctatus* were grouped into three genetically distinct groups (S2 Fig), one containing the populations of Aoos and Sarantaporos Rivers, another the populations of Aliakmonas, Axios and Pineios (Thessaly) Rivers, and the third the populations of Strymon River. The genetic distances between those groups were greater than 0.0200, while the maximum distance within each group did not exceed 0.0100 (S5 Table). Therefore, the specimens originally identified as *A*. *bipunctatus* could be assigned to other species. The populations from Aoos and Sarantaporos Rivers exhibited mean genetic distance equal to 0.0020 and maximum genetic distance equal to 0.0080 from *Alburnoides prespensis* (Karaman, 1924) and thus could be assigned to *A*. *prespensis* (which is found in Mikri Prespa and Megali Prespa Lakes). The close genetic relationship of the *Alburnoides* populations from these two rivers with the ones found in the two Prespa Lakes, is an argument in favor of the inclusion of the Prespa Lakes in the same biogeographical region as the river Aoos [11]. Based on the genetic distances, the populations of Aliakmonas, Axios and Pineios (Thessaly) Rivers could be incorporated into a distinct species, which has already been described under the name *Alburnoides thessalicus* Stephanidis, 1950 and which is also supported by Geiger et al. [40]. Additionally, our results are in accordance with the revaluation of the Strymon River population by Geiger et al. [40] under the name *Alburnoides strymonicus* Chichkoff, 1940. A very important finding is that all the studied Greek populations originally identified as *A*. *bipunctatus* exhibited genetic distance greater than 0.0200 from European populations (S5 Table), which means that, genetically, none of the Greek populations studied can be assigned to *A*. *bipunctatus*. These populations could be described as three other, separate *Alburnoides* species and other *Alburnoides* populations should be investigated as representatives of the species *A*. *bipunctatus* in Greece. The maximum distance within each group (0.0100) is much lower than the maximum intraspecific distance (0.0180) found for other species of the genus *Alburnoides* [69], reinforcing the argument that each group could consist a separate species.

#### Species of the genus *Cobitis*

On the phylogenetic tree of the genus *Cobitis*, the population of *Cobitis strumicae* Karaman, 1955 from Strymon River is grouped on a separate clade from the rest of *C*. *strumicae* (S3 Fig). The maximum genetic distance in either of these two groups is 0.0070 (S6 Table), while the mean distance between the groups is 0.0210 and the minimum distance between the groups is 0.0200. According to the genetic distances, the samples from Strymon River, originally identified as *C*. *strumicae*, could constitute a species separate both from *C*. *strumicae* and *C*. *punctilineata*, which is also found in Strymon River.

#### Species of the genus *Gobio*

Regarding the genus *Gobio*, the populations of *Gobio bulgaricus* Drensky, 1926 from Axios River and Doirani Lake (S4 Fig) exhibited maximum genetic distance between them equal to 0.0030, while the minimum distance from the other population of *G*. *bulgaricus* was 0.0440 (S7 Table). Therefore, those two populations could be separated from *G*. *bulgaricus* and designated as a separate species. The possibility of the Axios River population consisting a separate species has been previously mentioned by Barbieri et al. [9] and our genetic data support this. The fact that the minimum genetic distance of these populations from the other populations of *G*. *bulgaricus* was 5.5 times greater than the maximum genetic distance in either group, means that the separation of *G*. *bulgaricus* into two species could be genetically verified.

#### Species of the genus *Pelasgus*

The population of *Pelasgus* sp. (S5 Fig) from Kalamas River exhibited a maximum intra-population genetic distance equal to 0.0020, while the corresponding value for the population of Acheron River was 0.0030. The minimum distance between the two populations was 0.0330 (S8 Table), while the rest of the *Pelasgus* sp. specimens had a minimum genetic distance from the populations of Kalamas River and Acheron River equal to 0.0200 and 0.0320, respectively. Therefore, the populations of *Pelasgus* sp. from Kalamas and Acheron Rivers could be classified as two different species, since there was a clear barcoding gap between them.

#### Species of the genus *Telestes*

The populations of the *Telestes* genus from Acheloos, Alfeios, Evinos and Pineios (Peloponnese) Rivers, which were originally identified as *Telestes pleurobipunctatus* (Stephanidis, 1939) (S6 Fig), also exhibited genetic distances greater than 0.0200 (S9 Table). More specifically, the population from Acheloos River had a maximum intra-group distance of 0.0000, while its minimum distance from the other *Telestes* specimens was 0.0200, thus the Acheloos population could constitute a separate species. Additionally, the maximum genetic distance among the populations from Alfeios, Evinos and Pineios (Peloponnese) Rivers was 0.0150 and the minimum and mean distance of these population from the other *T*. *pleurobipunctatus* populations were 0.0170 and 0.0230 respectively (S9 Table). Based on that, the populations of Pineios (Peloponnese), Evinos and Alfeios Rivers could constitute another separate species, whose existence in the Alfeios and Pineios (Peloponnese) Rivers has been mentioned before [70,71], under the name *Telestes alfiensis* (Stephanidis, 1971). DNA barcoding seems to confirm the existence of this species, while at the same time it places the population of Evinos River in it.

### 3.4 Species with mean genetic distance lower than 0.0200

In nine cases species exhibited interspecific genetic distance lower than 0.0200 or a group of one species was grouped with a different species on the phylogenetic tree. These cases regarded the genera *Alburnus*, *Barbus*, *Cobitis*, *Rhodeus*, *Salmo* and *Squalius*.

#### Species of the genus *Alburnus*

The species *Alburnus thessalicus* Stephanidis, 1950 and *Alburnus macedonicus* Karaman, 1928 (S7 Fig) had a maximum genetic distance of 0.0070 (S10 Table) and they shared one haplotype, while the minimum distance between the other species of the genus was found equal to 0.0140. At the same time, the maximum distance between two other species of this genus, the species *Alburnus volviticus* Freyhof & Kottelat, 2007 and *Alburnus vistonicus* Freyhof & Kottelat, 2007 was 0.0050. These species exhibited very low interspecific distance and could not be genetically distinguished. It should be noted here that previously the *Alburnus* population from Filiouris River was not classified as *A*. *vistonicus* with certainty [9,72], however the results of the present study seem to strongly place this population in *A*. *vistonicus*. Based on the results of the genetic analysis, the two couples of *Alburnus* species mentioned above could be merged into two single species. However, since other studies have observed low genetic distance between some species of the genus *Alburnus* [73,74], further study of these species through nuclear or faster evolving genetic markers could help define their status more accurately.

During analysis of the samples identified as *Alburnus* sp. Volvi, a group from Strymon River (BOLD accession numbers: GFFB311-18—GFFB316-18) exhibited mean genetic distance lower than 0.0200 from *A*. *thessalicus* and *A*. *macedonicus*, and at the same a barcoding gap from these two species and the rest of the *Alburnus* sp. Volvi samples (S11 Table). Based on the genetic distances, while *Alburnus* sp. Volvi seems to be clearly differentiated from *A*. *thessalicus* and *A*. *macedonicus*, this certain group seems to be intermediary between them.

#### Species of the genus *Barbus*

In the genus *Barbus*, the specimens belonging to *Barbus euboicus* Stephanidis, 1950 were grouped together on the phylogenetic tree, but as a subclade of a *Barbus peloponnesius* Valenciennes, 1842 clade (S8 Fig). The maximum genetic distance between the two species was 0.0110, the maximum intraspecific distance was 0.0160 (S10 Table) and the two species did not have any haplotypes in common. Despite the low genetic distance of *B*. *euboicus* from *B*. *peloponnesius*, it was genetically distinct from all studied *B*. *peloponnesius* populations, which means that while *B*. *euboicus* could be genetically identified, however the genetic distances did not support its status as a separate species. These two species belong to different biogeographical regions, so they are in need of further study in order to clarify their relationship and investigate if translocations have taken place.

#### Species of the genus *Cobitis*

For the genus *Cobitis* (S3 Fig) the values of minimum genetic distance between its species (S10 Table) were among the highest for species of the same genus, meaning that these species were clearly separated. High values of interspecific genetic distance for the genus *Cobitis* have been observed in several studies [75,76]. The only exception are the species *Cobitis hellenica* Economidis & Nalbant, 1996 and *Cobitis arachthosensis* Economidis & Nalbant, 1996 for which the maximum genetic distance was 0.0070 and the minimum 0.0000. The lack of significant genetic differentiation between these two species could be attributed to the small distance separating the rivers in which they are found, which could mean that the species are still in contact. The minimum genetic distances between the other species of the genus range from 0.0550 to 0.1270, while the minimum genetic distance of these two species from the other species of the genus was 0.0860. That means the species *C*. *hellenica* and *C*. *arachthosensis*, with such a low maximum genetic distance, are more likely to consist a single species.

#### Species of the genus *Rhodeus*

In the genus *Rhodeus*, which included two species, *Rhodeus amarus* (Bloch, 1782) and *Rhodeus meridionalis* Karaman, 1924, specimens belonging to different species were genetically closer to each other than specimens of the same species (S9 Fig) Additionally, the maximum genetic distance was 0.0220 (S10 Table), which means that the threshold of 0.0200 is just barely surpassed. The morphological identification of these two species is particularly difficult and it has been claimed that their distinction is mainly based on genetic analysis [72]. However, both the present and a previous study [40] failed to detect genetic differentiation that could allow the identification of the species. The splitting of *Rhodeus* into two species is recent [77] and introgression between species of the genus has been observed [40,78]. All the above demonstrate that *Rhodeus* is a genus that needs more thorough investigation before reaching any conclusions. However, since the two species cannot be distinguished genetically and their morphological differences are almost imperceptible, their status as separate species is questioned.

#### Species of the genus *Salmo*

All individuals of the genus *Salmo* exhibited very low interspecific distance and could be distinguished only by morphological characters. The maximum genetic distance across all specimens was 0.0080, with the mean distance being 0.0040 and the maximum distance in a single species being 0.0070 (S10 Table). The two samples of *Salmo farioides* Karaman, 1938 from the Evinos and Arachthos Rivers exhibited haplotypes that were also found in *Salmo peristericus* Karaman, 1938, while the specimens of *Salmo* sp. from Nestos River were not grouped together on the phylogenetic tree but were distributed among the groups containing the other species (*S*. *farioides*, *S*. *peristericus*, *Salmo lourosensis* Delling, 2011) (S10 Fig). The fact that the species of the genus *Salmo* have diverged recently [79], combined with the translocations of specimens that took place in past decades in Greece [80] and the ability of these species to hybridize [81], perplex the efficacy of DNA barcoding identification.

#### Species of the genus *Squalius*

Regarding the genus *Squalius*, the Aoos River population has been assigned to *Squalius platyceps* Zupančič, Marić, Naseka & Bogutskaya, 2010 by some researchers [40,82], while others describe it as *Squalius* sp. *Aoos* Kottelat & Freyhof, 2007 [9,72]. On the phylogenetic tree the specimens of *Squalius* sp. *Aoos* and *S*. *platyceps* (all of which originated from the Aoos River) were grouped together with the species *Squalius prespensis* Fowler, 1977 (S11 Fig). The maximum genetic distance found between specimens of this group was 0.0030 (S10 Table), while the minimum distance between the other species of the genus ranged from 0.0150 to 0.0930. This means the separation of the Aoos River population from *S*. *prespensis* is not genetically justified. The genetic similarity of the *Squalius* populations from Aoos River and Mikri Prespa and Megali Prespa Lakes is yet another argument in favor of grouping these locations in the same biogeographical region, the same as in the case of the *Alburnoides* populations of these locations.

According to Barbieri et al. [9], *Squalius moreoticus* (Stephanidis, 1971) is morphologically indistinguishable from *Squalius peloponensis* (Valenciennes, 1844). The available DNA barcodes for this species originate from Vouraikos River and their genetic distance from the available samples of *S*. *peloponensis* is less than 0.0200 (S10 Table), while the two species share haplotypes. *S*. *moreoticus* is endemic in Stymphalia Lake, while its presence in Vouraikos river was mentioned by Kottelat and Freyhof [72]. The genetic data, however, seem to suggest that the *Squalius* population of Vouraikos River should be classified as *S*. *peloponensis*. To clarify the status of *S*. *moreoticus* and its relationship with *S*. *peloponensis*, samples from Stymphalia Lake need to be analyzed.

### 3.5 Reassignment of populations to species

The specimens of *B*. *peloponnesius* from Arachthos River and certain specimens of *B*. *peloponnesius* from Kalamas and Acheloos Rivers (S8 Fig) exhibit maximum genetic distance from *Barbus prespensis* Karaman, 1924 equal to 0.0150, which is also the value of the maximum distance within either group. Excluding the samples of *B*. *peloponnesius* mentioned above, the minimum genetic distance of *B*. *peloponnesius* from *B*. *prespensis* is 0.0260, which means the two species were clearly differentiated. The minimum genetic distance between the other species of the genus was at least 0.0310 and the maximum intraspecific distance found in them was 0.0060. This means that based on the genetic distances, the samples of *B*. *peloponnesius* from Arachthos River and certain specimens of *B*. *peloponnesius* from Kalamas and Acheloos Rivers could be assigned to *B*. *prespensis*. The expansion of *B*. *prespensis* beyond the Prespa Lakes [83] and its presence in Kalamas River [40] have been described before and our findings not only seem to confirm this, but also indicated that *B*. *prespensis* is present in Arachthos River instead of *B*. *peloponnesius* and that the two species are sympatric in Kalamas and Acheloos Rivers. Thus, this case seems to be about reassigning certain populations of one species to another and not about questioning the status of these species as separate ones.

### 3.6 Automatic Barcode Gap Discovery (ABGD)

The ABGD analysis was also used as a supplementary methodology to species delimitation when using molecular data. The ABGD analysis calculated the Barcode Gap distance equal to 0.0090. The samples were grouped into 77 groups, provided in S12 Table. Generally, ABGD grouping was in accordance with the results obtained when using the threshold of 0.0200 criteria, verifying the existence of cryptic diversity in both cases. Differences are found only in seven cases, where samples considered as one group by the previous analyses, were split in two separate groups by ABGD. Six of the cases are groups of the same species that exhibit a barcoding gap, but the mean distance between the two groups is lower than 0.0200 and this is the reason they differ from the previous approach. The genetic distances for the groups of these seven cases are presented in S13 Table. More specifically, of the seven cases that differ between ABGD and the previous approach:

Two cases concerned species represented by only two or three samples and they were split by ABGD in two groups. These are possibly artifacts of the small sample size, and no conclusions can be drawn before more samples are analyzed. This is the case for i) the species *Cobitis meridionalis* Karaman, 1924 from Megali Pespa Lake (S3 Fig) and ii) the species *Pelasgus prespensis* (Karaman, 1924) from Megali Prespa and Mikri Prespa Lakes (S5 Fig).

The rest of the cases can act as indications for isolated groups: i) Samples of *Barbus peloponnesius* from Kalamas River were considered a separate group from samples of *Barbus peloponnesius* from Alfeios, Vouraikos and Acheloos Rivers and *Barbus euboicus* (S8 Fig). ii) *Luciobarbus albanicus* (Steindachner, 1870), was separated into two groups, one containing the samples from Arachthos, Kalamas and one sample from Acheloos Rivers and the other containing the samples from Pineios (Peloponnese) and Acheloos Rivers (S12 Fig). iii) In *Pachychilon macedonicum* (Steindachner, 1892), a single sample from Pineios (Thessaly) River, was considered a separate group from all the other samples of the species (S13 Fig). iv) The samples of the genus *Rhodeus* were separated in four groups, corresponding exactly to the four clades of the *Rhodeus* phylogenetic tree (S9 Fig). v) The samples of *Telestes pleurobipunctatus* from Kalamas and Acheron Rivers were considered a separate group from the ones from Arachthos and Louros Rivers (S6 Fig).

In the aforementioned cases there is a barcode gap, but the small genetic distances and the absence of significant morphological differentiation prevent us from proposing the split into separate species. ABGD has been found to perform well for mitochondrial DNA [84] and this is also confirmed by our results. However, it is considered more useful as a tool for the delineation of species, not by itself, but as part of a broader, integrative taxonomic approach [85]. The discrepancies in species delineation between ABGD and the approach using the barcode gap and a threshold of genetic distance could be resolved by the analysis of more samples (especially in cases where small sample sizes were analyzed), the use of different molecular markers and the combination of genetic data with other taxonomic approaches like biogeography and morphology.

### 3.7 Regions with region specific haplotypes

During the analyses, 22 populations exhibited region-specific haplotypes. These cases were listed in Table 3. In most of these cases, genetic distances do not justify the designation of these populations into separate species; they simply seem to be isolated populations. Based on that, if a sample belongs to certain species, we can define whether it originates from one of those regions. This demonstrates the potential of DNA barcoding to go a step deeper from the identification of species. This means that if a sufficient number of individuals from these species has been analyzed, (in order to avoid errors due to inadequate sampling), the sequence of the COI gene could potentially be used not only to assign an individual to species level, but also to track species geographic origin.

**Table 3:** Populations of species with region specific haplotypes.

Species	Region
*Alburnoides thessalicus*	Pineios River (Thessaly)
*Alburnoides prespensis*	Prespes Lakes
*Barbus euboicus*	Euboea
*Barbus peloponnesius*	Alfeios River, Vouraikos River
*Cobitis strumicae*	Strymon River
*Gasterosteus sp*.	Louros River, Acheron River, Aris River
*Gobio bulgaricus*	Filiouris River, Evros River
*Pachychilon macedonicum*	Doirani Lake
*Pelasgus sp*.	Kalamas River, Acheron River, Eurotas River, Alfeios River
*Rhodeus amarus*	Kompsatos River
*Salmo farioides*	Aoos River
*Squalius sp*.	Euboea
*Telestes pleurobipunctatus*	Acheloos River, Alfeios River
*Vimba melanops*	Axios River

## 4. Conclusion

The constant increase of available DNA barcodes enables the continuous improvement of DNA barcoding efficiency and the resolution of previously conflicting or unresolved cases. DNA barcoding combined with traditional classification methods provides a powerful tool for the identification of species, which has already led to many taxonomic revisions and will continue to do so, as its use becomes more widespread.

The results of the present study will contribute to the recent efforts to revise the taxonomy of Greek fish species and clarify their phylogenetic relationships, expanding our knowledge on the biodiversity of Greece and will thus contribute to the efforts to conserve it. At the same time, we hope that the new sequences produced will enrich, to an extent, the DNA barcode databases and prove useful for the overall effort to accurately identify species through genetic markers.

For all the cases of low interspecific distance and lack of barcoding gap, we propose further study in order to determine whether species separation is justified or whether they could be characterized as synonym species and could possibly be merged. A large-scale sampling, including regions not covered in past studies, could help to acquire more DNA barcodes for each species and at the same time clarify if the unclear cases concern only the populations from certain regions or the entire expansion range of these species. Additionally, in cases of closely related or sympatric species, the use of nuclear markers could be used to identify hybrids and introgression between species. It should be noted that any further study of these cases should be a collaboration of both geneticists and ichthyologists, as well as biogeography experts, since their combined effort will make it be easier to produce results and reach safer conclusions. Since the taxonomic status of many Greek freshwater fish species is still unresolved [9], a future revision for some of them, that takes into account genetic information, is bound to improve the accuracy of DNA barcoding.

## Supporting information

S1 Fig Phylogenetic tree (ML) of all studied species. Sequences of the same species have been collapsed. On each clade, the bootstrap value is given.
(PNG)

S2 Fig Phylogenetic tree (ML) of the genus *Alburnoides*. Samples grouped together during the analyses are marked with the same color. The circle denotes the sequences recovered from BOLD, while the circular disk denotes the sequences produced from the present study. For each sample the species, the area of origin and the BOLD accession number are given.
(PNG)

S3 Fig Phylogenetic tree (ML) of the genus *Cobitis*. Samples grouped together during the analyses are marked with the same color. The circle denotes the sequences recovered from BOLD, while the circular disk denotes the sequences produced from the present study. For each sample the species, the area of origin and the BOLD accession number are given. The groups split by ABGD are signified by brackets.
(PNG)

S4 Fig Phylogenetic tree (ML) of the genus *Gobio*. Samples grouped together during the analyses are marked with the same color. The circle denotes the sequences recovered from BOLD, while the circular disk denotes the sequences produced from the present study. For each sample the species, the area of origin and the BOLD accession number are given.
(PNG)

S5 Fig Phylogenetic tree (ML) of the genus *Pelasgus*. Samples grouped together during the analyses are marked with the same color. The circle denotes the sequences recovered from BOLD, while the circular disk denotes the sequences produced from the present study. For each sample the species, the area of origin and the BOLD accession number are given. The groups split by ABGD are signified by brackets.
(PNG)

S6 Fig Phylogenetic tree (ML) of the genus *Telestes*. Samples grouped together during the analyses are marked with the same color. The circle denotes the sequences recovered from BOLD, while the circular disk denotes the sequences produced from the present study. For each sample the species, the area of origin and the BOLD accession number are given. The groups split by ABGD are signified by brackets.
(PNG)

S7 Fig Phylogenetic tree (ML) of the genus *Alburnus*. Samples grouped together during the analyses are marked with the same color. The circle denotes the sequences recovered from BOLD, while the circular disk denotes the sequences produced from the present study. For each sample the species, the area of origin and the BOLD accession number are given.
(PNG)

S8 Fig Phylogenetic tree (ML) of the genus *Barbus*. Samples grouped together during the analyses are marked with the same color. The circle denotes the sequences recovered from BOLD, while the circular disk denotes the sequences produced from the present study. For each sample the species, the area of origin and the BOLD accession number are given. The groups split by ABGD are signified by brackets.
(PNG)

S9 Fig Phylogenetic tree (ML) of the genus *Rhodeus*. Samples grouped together during the analyses are marked with the same color. The circle denotes the sequences recovered from BOLD, while the circular disk denotes the sequences produced from the present study. For each sample the species, the area of origin and the BOLD accession number are given. The groups split by ABGD are signified by brackets.
(PNG)

S10 Fig Phylogenetic tree (ML) of the genus *Salmo*. Samples grouped together during the analyses are marked with the same color. The circle denotes the sequences recovered from BOLD, while the circular disk denotes the sequences produced from the present study. For each sample the species, the area of origin and the BOLD accession number are given.
(PNG)

S11 Fig Phylogenetic tree (ML) containing certain species of the genus *Squalius*. Samples grouped together during the analyses are marked with the same color. The circle denotes the sequences recovered from BOLD, while the circular disk denotes the sequences produced from the present study. For each sample the species, the area of origin and the BOLD accession number are given.
(PNG)

S12 Fig Phylogenetic tree (ML) of the genus *Luciobarbus*. Samples grouped together during the analyses are marked with the same color. The circle denotes the sequences recovered from BOLD, while the circular disk denotes the sequences produced from the present study. For each sample the species, the area of origin and the BOLD accession number are given. The groups split by ABGD are signified by brackets.
(PNG)

S13 Fig Phylogenetic tree (ML) of the genus *Pachychilon*. Samples grouped together during the analyses are marked with the same color. The circle denotes the sequences recovered from BOLD, while the circular disk denotes the sequences produced from the present study. For each sample the species, the area of origin and the BOLD accession number are given. The groups split by ABGD are signified by brackets.
(PNG)

S1 Table Samples analyzed and submitted to BOLD during the study. For each sample are given the species to which it belongs, the BOLD accession number, the ID number with which the sample can be found in the sample collection and the area of origin.
(XLSX)

S2 Table Species studied during the study. For each species, the number of sequences produced during the present study and the number of sequences retrieved from BOLD are given.
(XLSX)

S3 Table Sequences retrieved from BOLD. For each genus, the BOLD accession number and the area origin of each sequence is given.
(XLSX)

S4 Table DNA barcoding success for the studied species. For each species it is reported if it can be successfully identified by DNA barcoding, the mean intra-specific genetic distance, the maximum intra-specific genetic distance, and the minimum genetic distance from its closest-related species of the same genus. In the parentheses is given the standard error of the genetic distance. Species marked with ^*a*^ are species with mean intra-specific genetic distance greater than 0.0200 and they are presented in more detail in part 3.3 of the manuscript. Species marked with ^b^ are species with mean genetic distance from their closest-related species of the same genus lower than 0.0200 and no barcoding gap and they are presented in more detail in part 3.4 of the manuscript. Species marked with ^c^ are species with mean genetic distance from their closest-related species of the same genus lower than 0.0200 but exhibiting a barcoding gap and they are presented in more detail in part 3.4 of the manuscript. In the columns reporting genetic distances from closest-related species of the same genus (columns 5 and 6), a dash (-) means the species was the only one studied from this genus.
(XLSX)

S5 Table Genetic distances between populations of the genus *Alburnoides* originally identified as *A*. *bipunctatus*. The names correspond to the areas of origin of each group. The mean genetic distance between the groups is given below the diagonal, the maximum distance within each group on the diagonal and the minimum distance between groups above the diagonal. In the parentheses is given the standard error.
(XLSX)

S6 Table Genetic distances between populations of the genus *Cobitis* originally identified as *C*. *strumicae*. The names correspond to the areas of origin of each group. The mean genetic distance between the groups is given below the diagonal, the maximum distance within each group on the diagonal and the minimum distance between groups above the diagonal. In the parentheses is given the standard error.
(XLSX)

S7 Table Genetic distances between populations of the genus *Gobio* originally identified as *G*. *bulgaricus*. The names correspond to the areas of origin of each group. The mean genetic distance between the groups is given below the diagonal, the maximum distance within each group on the diagonal and the minimum distance between groups above the diagonal. In the parentheses is given the standard error.
(XLSX)

S8 Table Genetic distances between populations of the genus *Pelasgus* originally identified as *Pelasgus spp*. The names correspond to the areas of origin of each group. The mean genetic distance between the groups is given below the diagonal, the maximum distance within each group on the diagonal and the minimum distance between groups above the diagonal. In the parentheses is given the standard error.
(XLSX)

S9 Table Genetic distances between populations of the genus *Telestes* originally identified as *T*. *pleurobipunctatus*. The names correspond to the areas of origin of each group. The mean genetic distance between the groups is given below the diagonal, the maximum distance within each group on the diagonal and the minimum distance between groups above the diagonal. In the parentheses is given the standard error.
(XLSX)

S10 Table Genetic distances between and within species with mean genetic distance lower than 0.0200. In parentheses, the standard error is given.
(XLSX)

S11 Table Genetic distances between the two groups of *Alburnus* sp. Volvi and the group *Alburnus thessalicus–Alburnus macedonicus*. The mean genetic distance between the groups is given below the diagonal, the maximum distance within each group on the diagonal and the minimum distance between groups above the diagonal. In the parentheses is given the standard error.
(XLSX)

S12 Table Results of the ABGD analysis. Each line corresponds to one group of samples, as defined by ABGD. For each group, the name of all the samples belonging to it are given.
(XLSX)

S13 Table Genetic distances of species divided by ABGD into additional groups. (XLSX)

S14 Table Genetic distances between groups of the genus *Rhodeus*, as defined by ABGD. The names correspond to the areas of origin of each group. The mean genetic distance between the groups is given below the diagonal, the maximum distance within each group on the diagonal and the minimum distance between groups above the diagonal. In the parentheses is given the standard error.
(XLSX)

S1 File Dataset used for the analyses. The dataset includes both sequences produced during the present study and sequences recovered from BOLD. The name of each sequence includes the genus and species of the sample, the location of origin and its BOLD accession number.
(FAS)
