# Supplementary figures and images for: DNA barcoding identification of Greek freshwater fishes
Source: PLoS One. 2022 Jan 26;17(1):e0263118. doi: 10.1371/journal.pone.0263118 (PMC8791500; doi:10.1371/journal.pone.0263118)

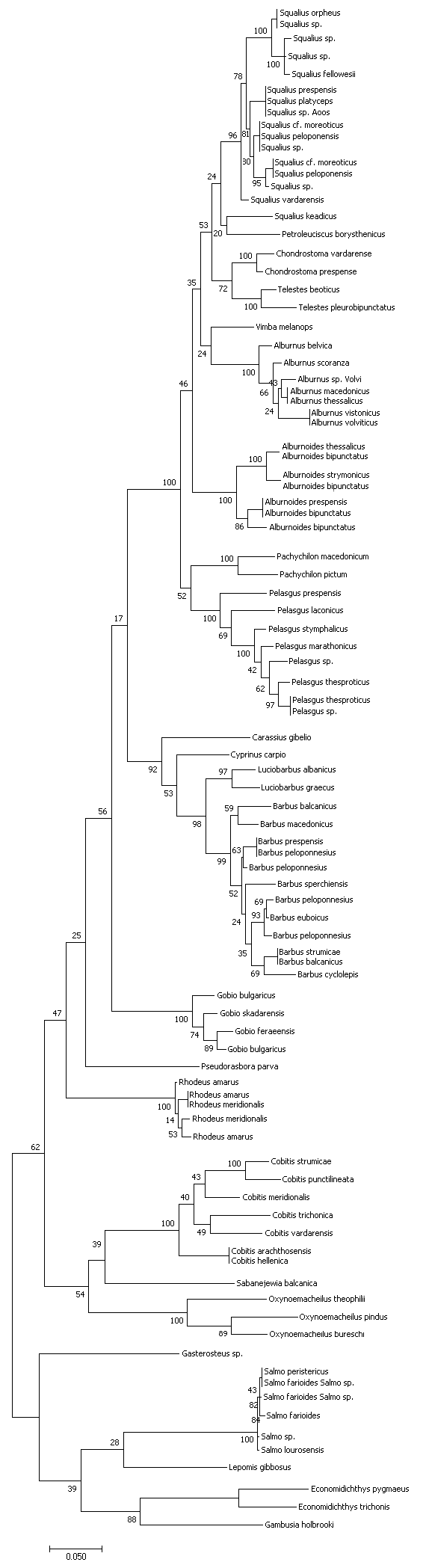

Supplement: S1 Fig — Sequences of the same species have been collapsed. On each clade, the bootstrap value is given. (PNG) [file pone.0263118.s001.png]

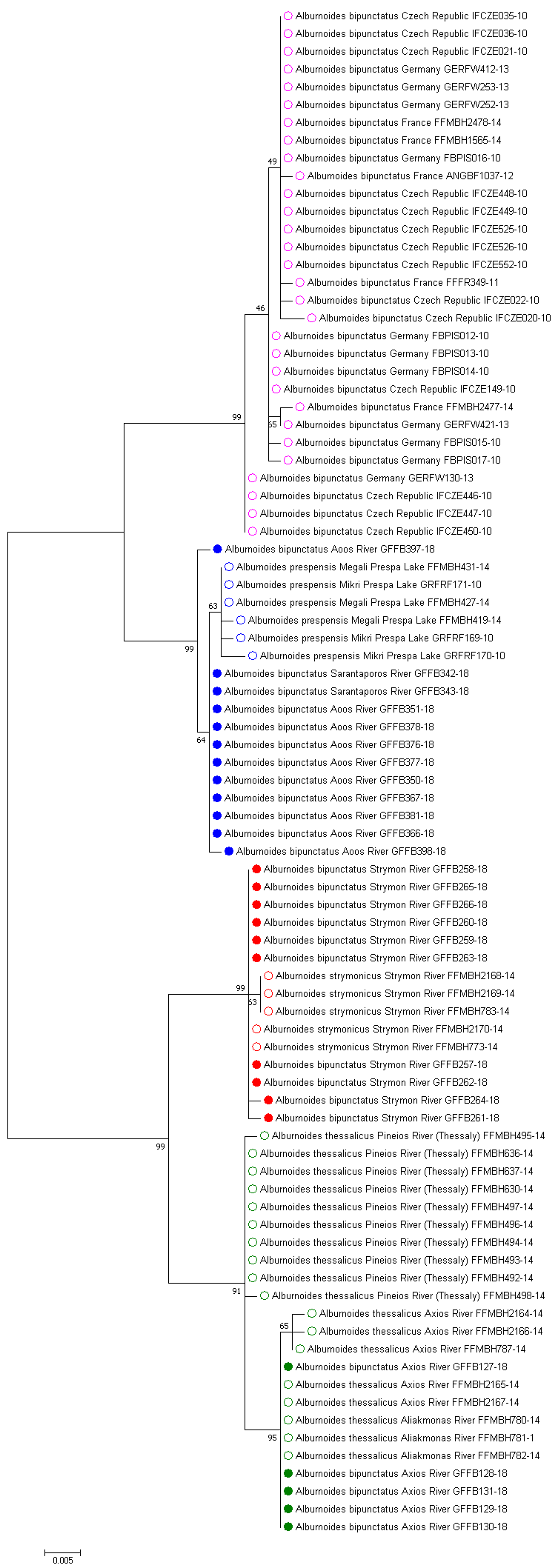

Supplement: S2 Fig — Samples grouped together during the analyses are marked with the same color. The circle denotes the sequences recovered from BOLD, while the circular disk denotes the sequences produced from the present study. For each sample the species, the area of origin and the BOLD accession number are given. (PNG) [file pone.0263118.s002.PNG]

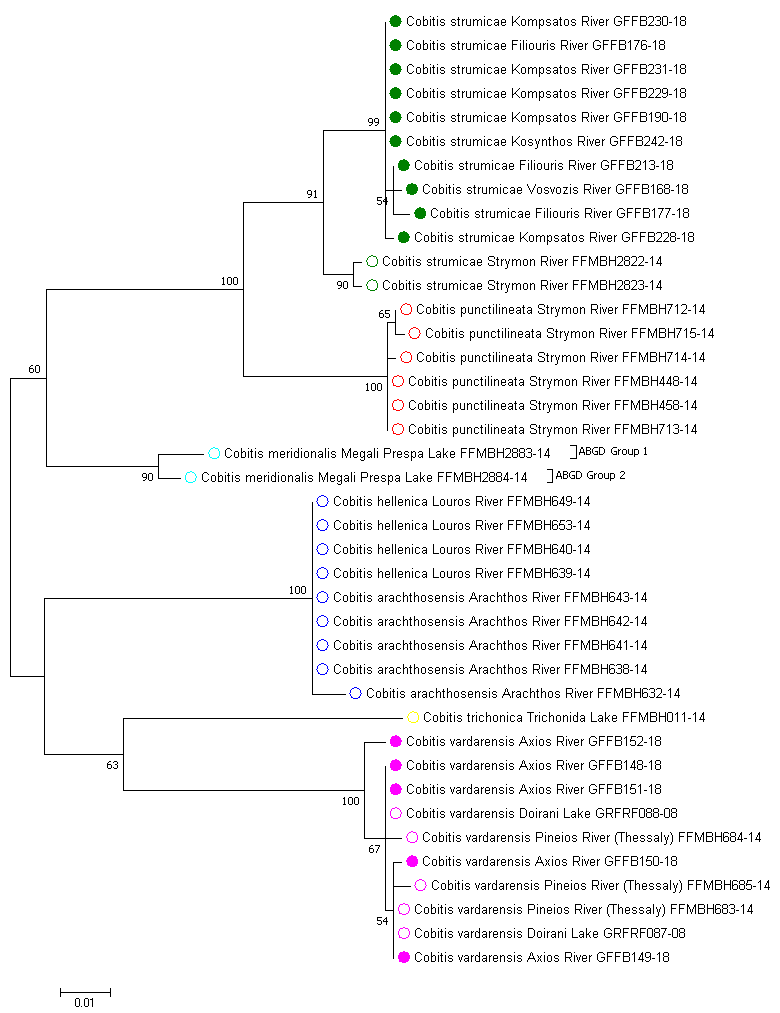

Supplement: S3 Fig — Samples grouped together during the analyses are marked with the same color. The circle denotes the sequences recovered from BOLD, while the circular disk denotes the sequences produced from the present study. For each sample the species, the area of origin and the BOLD accession number are given. The groups split by ABGD are signified by brackets. (PNG) [file pone.0263118.s003.png]

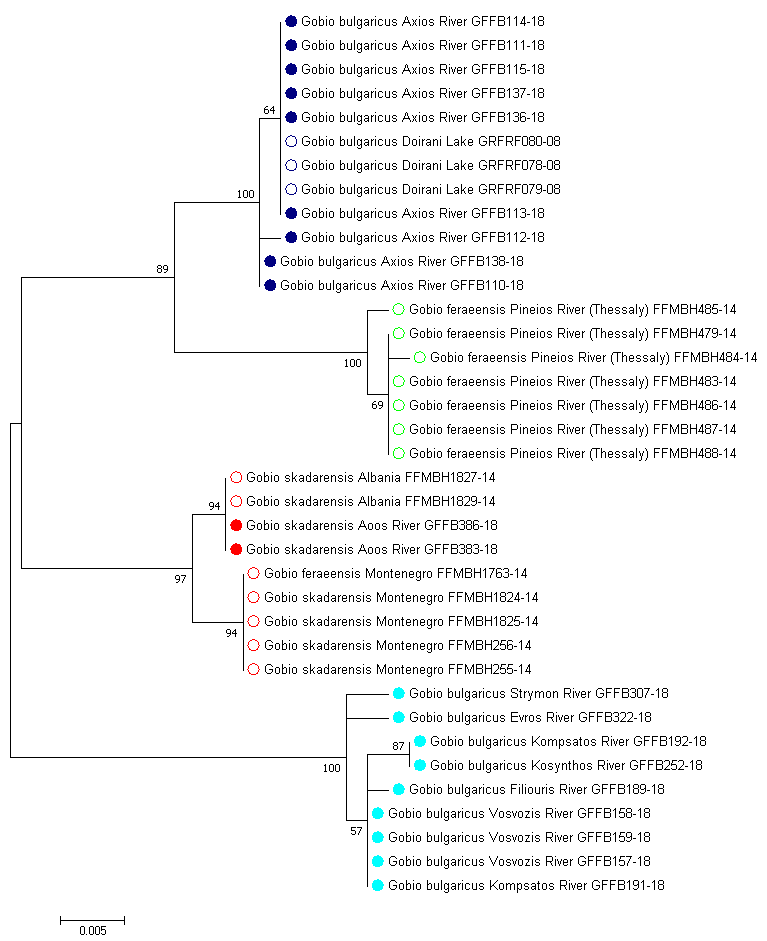

Supplement: S4 Fig — Samples grouped together during the analyses are marked with the same color. The circle denotes the sequences recovered from BOLD, while the circular disk denotes the sequences produced from the present study. For each sample the species, the area of origin and the BOLD accession number are given. (PNG) [file pone.0263118.s004.png]

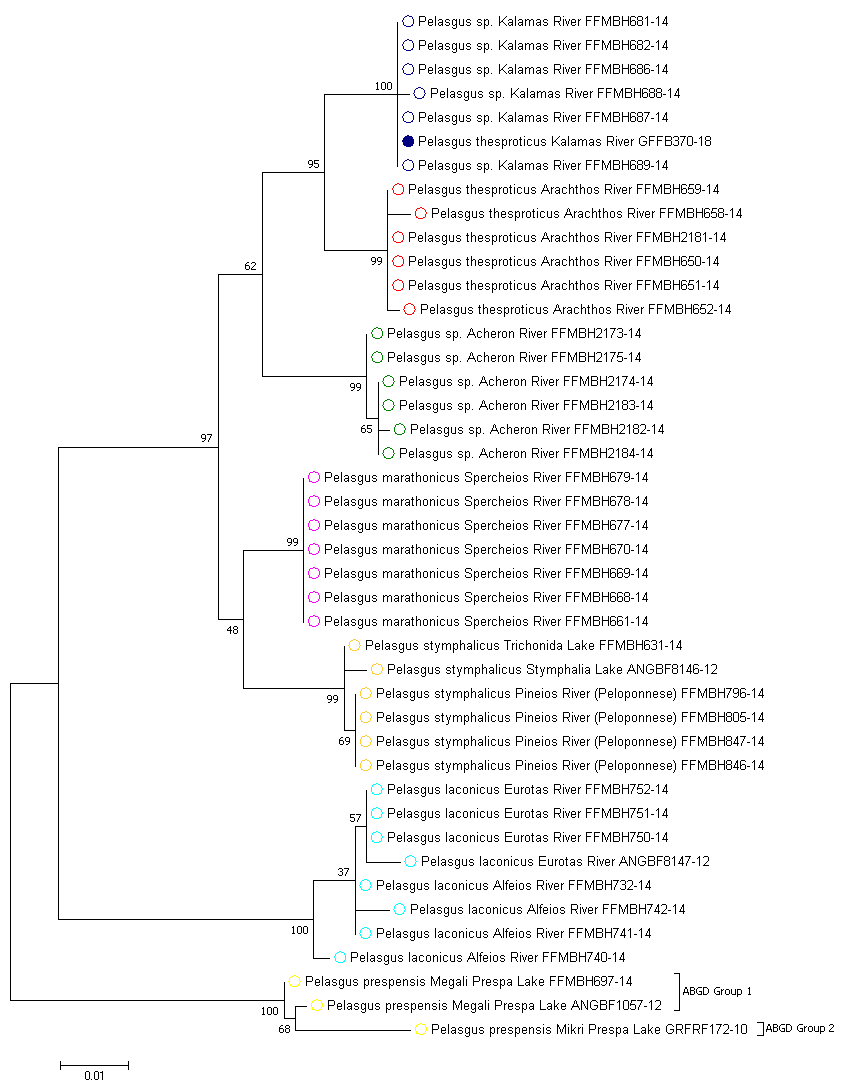

Supplement: S5 Fig — Samples grouped together during the analyses are marked with the same color. The circle denotes the sequences recovered from BOLD, while the circular disk denotes the sequences produced from the present study. For each sample the species, the area of origin and the BOLD accession number are given. The groups split by ABGD are signified by brackets. (PNG) [file pone.0263118.s005.png]

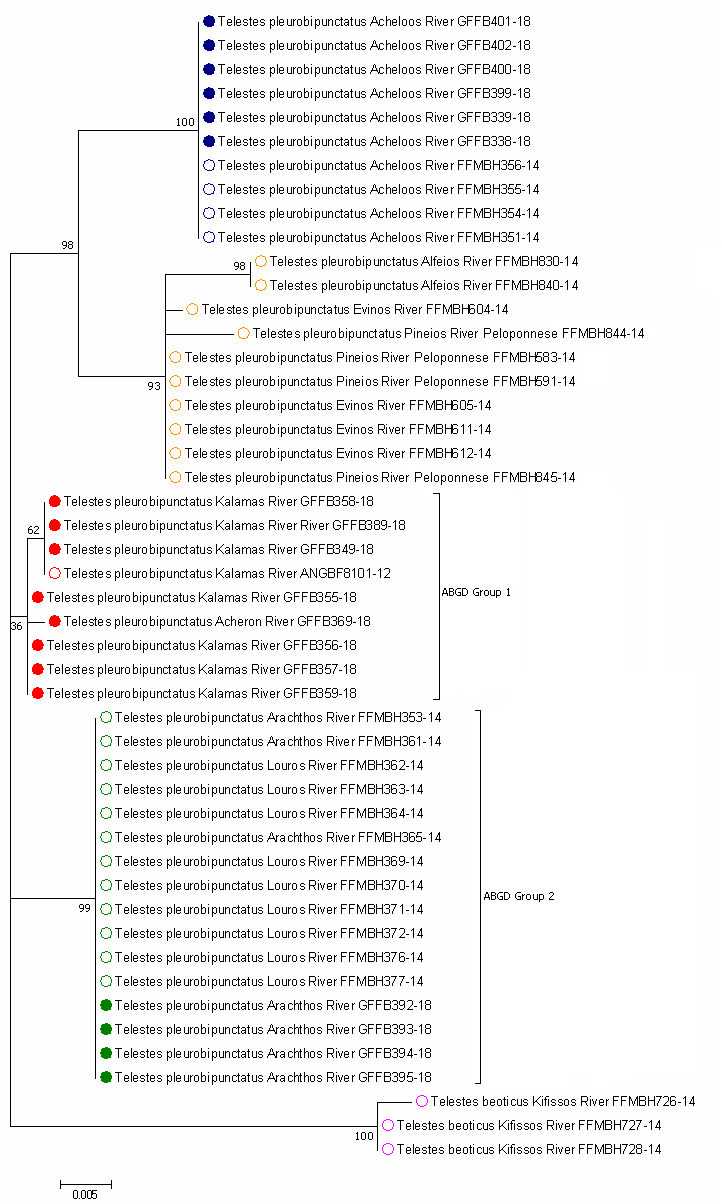

Supplement: S6 Fig — Samples grouped together during the analyses are marked with the same color. The circle denotes the sequences recovered from BOLD, while the circular disk denotes the sequences produced from the present study. For each sample the species, the area of origin and the BOLD accession number are given. The groups split by ABGD are signified by brackets. (PNG) [file pone.0263118.s006.png]

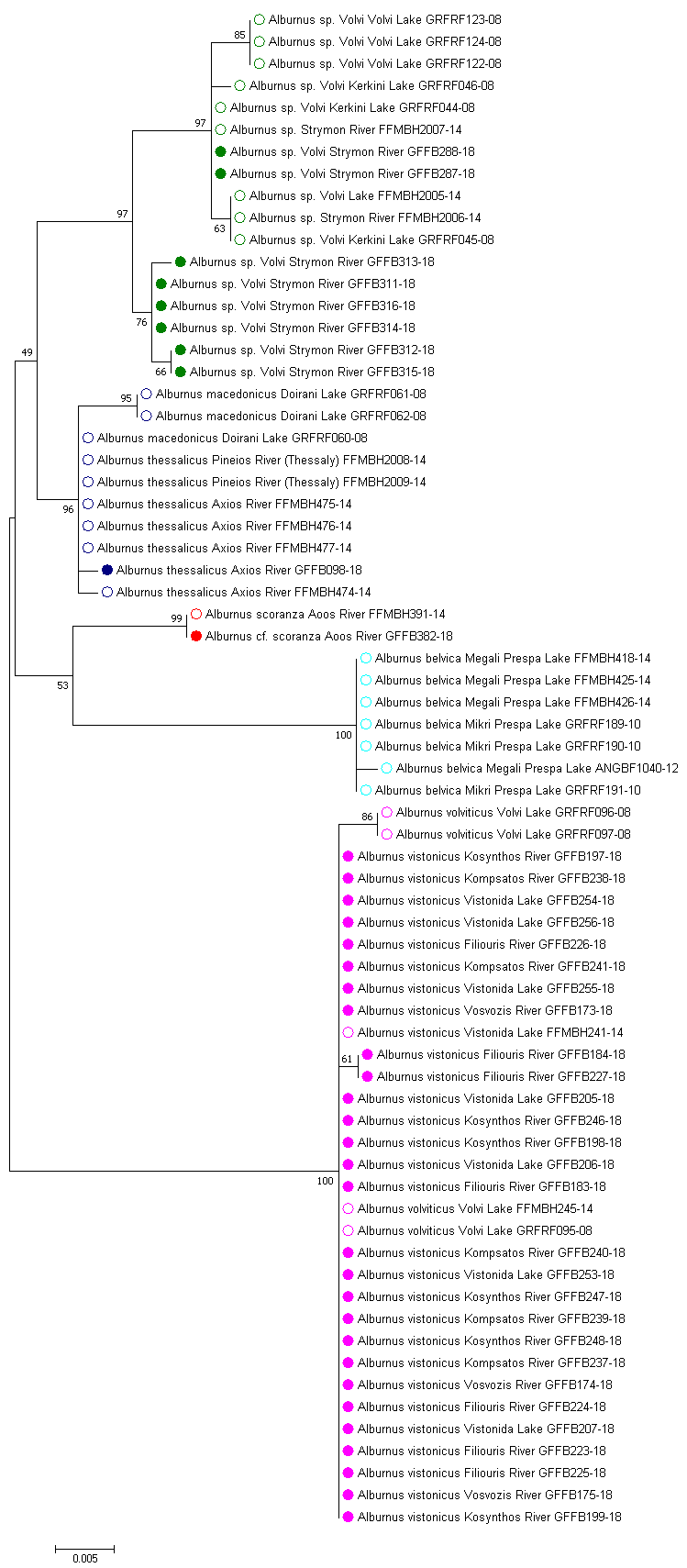

Supplement: S7 Fig — Samples grouped together during the analyses are marked with the same color. The circle denotes the sequences recovered from BOLD, while the circular disk denotes the sequences produced from the present study. For each sample the species, the area of origin and the BOLD accession number are given. (PNG) [file pone.0263118.s007.png]

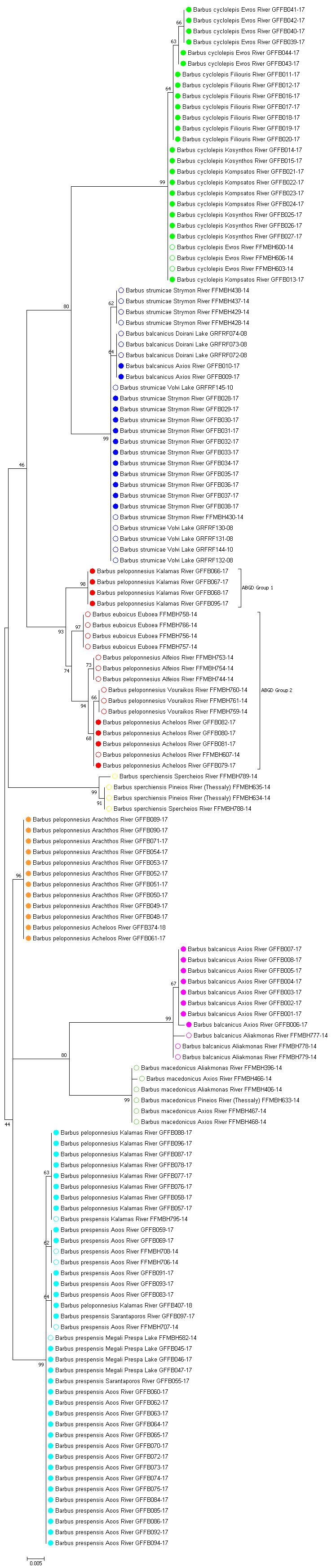

Supplement: S8 Fig — Samples grouped together during the analyses are marked with the same color. The circle denotes the sequences recovered from BOLD, while the circular disk denotes the sequences produced from the present study. For each sample the species, the area of origin and the BOLD accession number are given. The groups split by ABGD are signified by brackets. (PNG) [file pone.0263118.s008.png]

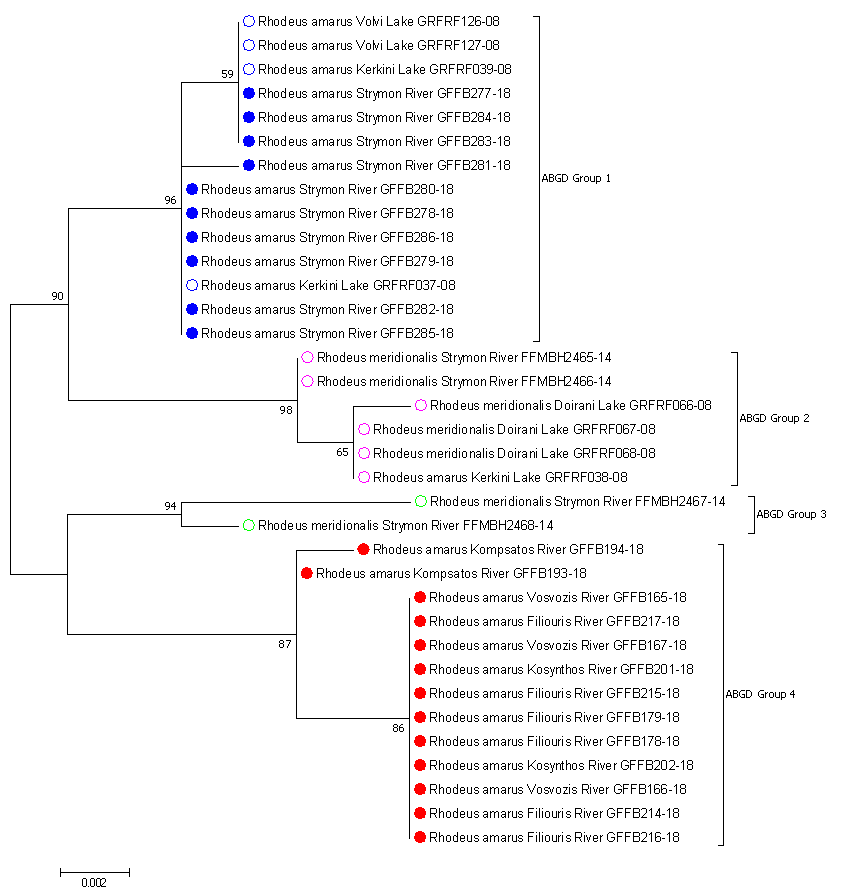

Supplement: S9 Fig — Samples grouped together during the analyses are marked with the same color. The circle denotes the sequences recovered from BOLD, while the circular disk denotes the sequences produced from the present study. For each sample the species, the area of origin and the BOLD accession number are given. The groups split by ABGD are signified by brackets. (PNG) [file pone.0263118.s009.PNG]

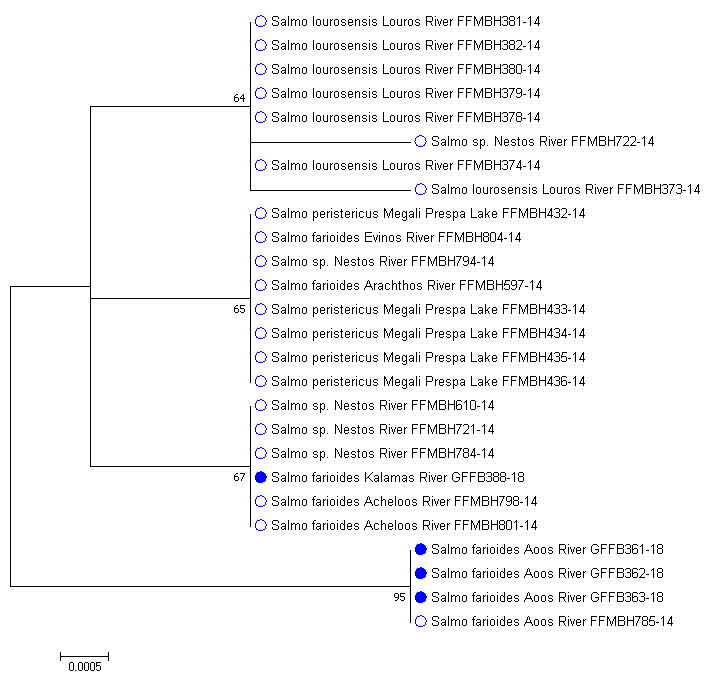

Supplement: S10 Fig — Samples grouped together during the analyses are marked with the same color. The circle denotes the sequences recovered from BOLD, while the circular disk denotes the sequences produced from the present study. For each sample the species, the area of origin and the BOLD accession number are given. (PNG) [file pone.0263118.s010.png]

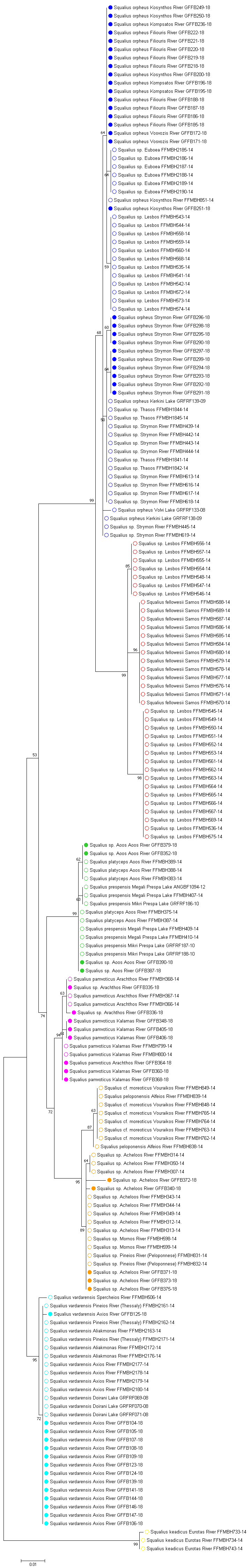

Supplement: S11 Fig — Samples grouped together during the analyses are marked with the same color. The circle denotes the sequences recovered from BOLD, while the circular disk denotes the sequences produced from the present study. For each sample the species, the area of origin and the BOLD accession number are given. (PNG) [file pone.0263118.s011.png]

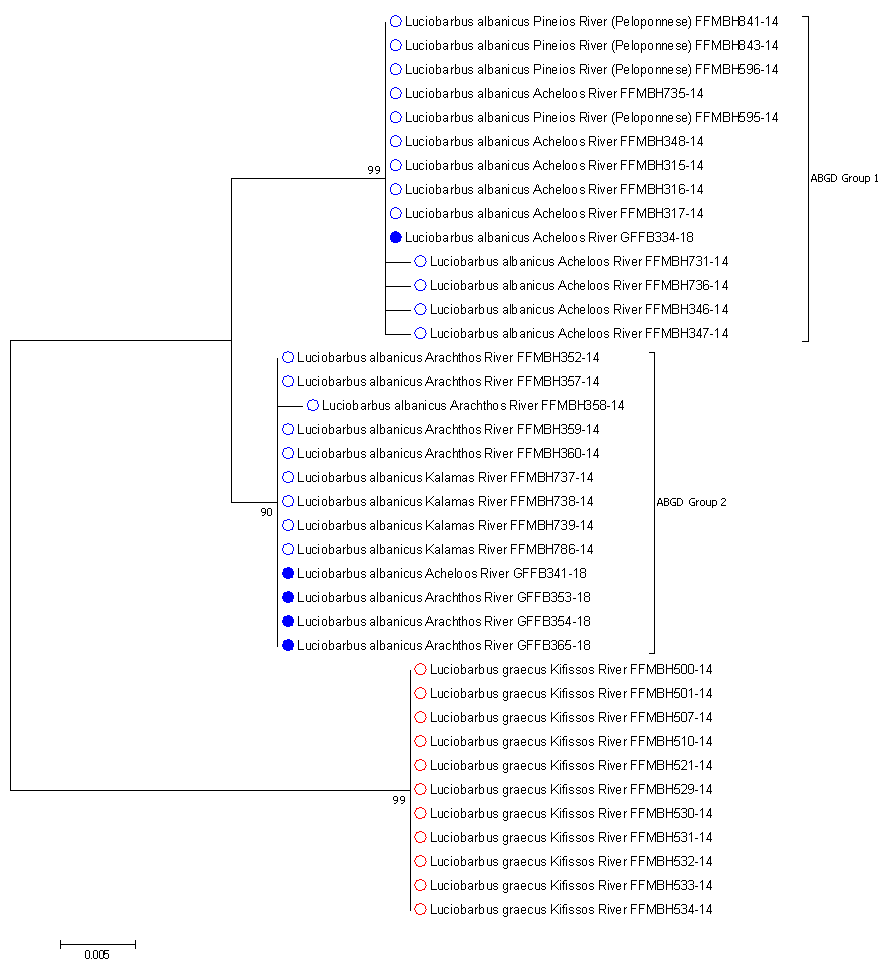

Supplement: S12 Fig — Samples grouped together during the analyses are marked with the same color. The circle denotes the sequences recovered from BOLD, while the circular disk denotes the sequences produced from the present study. For each sample the species, the area of origin and the BOLD accession number are given. The groups split by ABGD are signified by brackets. (PNG) [file pone.0263118.s012.png]

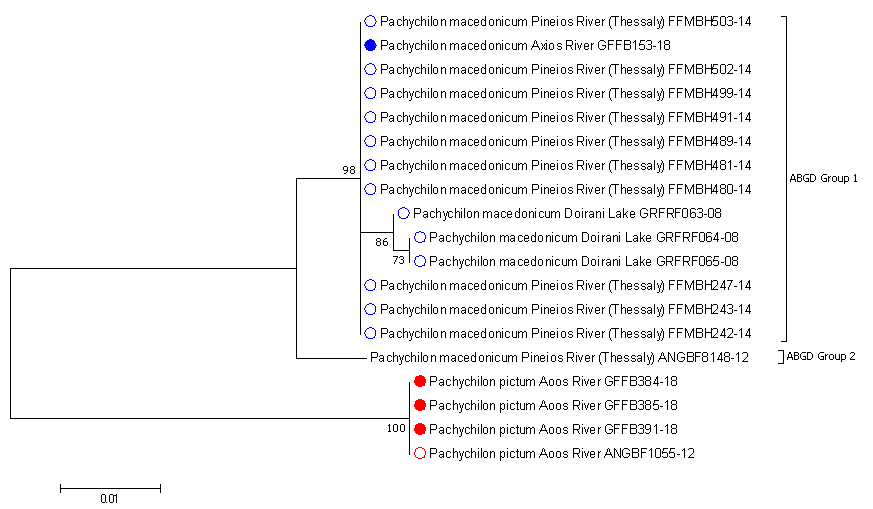

Supplement: S13 Fig — Samples grouped together during the analyses are marked with the same color. The circle denotes the sequences recovered from BOLD, while the circular disk denotes the sequences produced from the present study. For each sample the species, the area of origin and the BOLD accession number are given. The groups split by ABGD are signified by brackets. (PNG) [file pone.0263118.s013.png]
